# Shoulder and Scapular Function Before and After a Scapular Therapeutic Exercise Program for Chronic Shoulder Pain and Scapular Dyskinesis: A Pre–Post Single-Group Study

**DOI:** 10.3390/jpm15070285

**Published:** 2025-07-02

**Authors:** Ana S. C. Melo, Ana L. Soares, Catarina Castro, Ricardo Matias, Eduardo B. Cruz, J. Paulo Vilas-Boas, Andreia S. P. Sousa

**Affiliations:** 1 Centro de Investigação em Reabilitação (CIR), Escola Superior de Saúde, Instituto Politécnico do Porto, Rua Dr. António Bernardino de Almeida, 400, 4200-072 Porto, Portugal; ame@ess.ipp.pt (A.S.C.M.); 10210169@ess.ipp.pt (A.L.S.); 10210170@ess.ipp.pt (C.C.); 2 Centro de Investigação em Actividade Física, Saúde e Lazer (CIAFEL), Faculdade de Desporto, Universidade do Porto, Rua Dr. Plácido Costa, 91, 4200-450 Porto, Portugal; 3 Laboratório de Biomecânica do Porto (LABIOMEP), Universidade do Porto, Rua Dr. Plácido Costa, 91, 4200-450 Porto, Portugal; jpvb@fade.up.pt; 4 Centro Interdisciplinar de Investigação Aplicada em Saúde (CIIAS), Escola Superior de Saúde, Instituto Politécnico de Setúbal, Campus do IPS Estefanilha, 2914-503 Setúbal, Portugal; 5 Departamento de Física, Instituto de Biofísica e Engenharia Biomédica (IBEB), Faculdade de Ciências, Universidade de Lisboa, 1749-016 Lisboa, Portugal; rmatias@me.com; 6 Departamento de Fisioterapia, Escola Superior de Saúde, Instituto Politécnico de Setúbal, Campus do IPS Estefanilha, 2914-503 Setúbal, Portugal; eduardo.cruz@ess.ips.pt; 7 Centro de Investigação Integrada em Saúde (CHRC), Universidade Nova de Lisboa, 1169-056 Lisboa, Portugal; 8 Centro de Investigação, Formação, Inovação e Intervenção em Desporto (CIFI2D), Faculdade de Desporto, Universidade do Porto, Rua Dr. Plácido Costa, 91, 4200-450 Porto, Portugal

**Keywords:** biomechanical phenomena, pain, rehabilitation, scapula, superficial back muscles

## Abstract

**Background/Objectives**: Scapular adaptations have been associated with shoulder pain. However, conflicting findings have been reported after scapular-focused interventions. The present study aims to evaluate scapula-related outcomes before and after a scapular therapeutic exercise program. **Methods**: Eighteen adult volunteers with chronic shoulder pain participated in an 8-week scapular therapeutic exercise program that was personalized according to their pain condition and the presence of scapular dyskinesis. This program included preparation and warm-up, scapular neuromotor control, and strengthening and stretching exercises. Both self-reported (shoulder pain and function, psychosocial factors, and self-impression of change) and performance-based outcomes (scapular muscular stiffness and activity level, tridimensional motion, rhythm, and movement quality, measured while participants drank a bottle of water) were used for analysis. **Results**: After the intervention, participants presented reduced shoulder pain (*p* < 0.0001) and pain catastrophizing (*p* = 0.004) and increased shoulder function (*p* < 0.0001). Additionally, the participants presented changes in scapular winging (*p* < 0.0001 to *p* = 0.043), increased scapular downward rotation (*p* < 0.0001) and depression (*p* = 0.038), and decreased global movement smoothness (*p* = 0.003). These were associated with changes in serratus anterior activity (*p* = 0.016 to *p* = 0.035), decreased middle (*p* < 0.0001 to *p* = 0.002) and lower trapezius (*p* < 0.0001) and levator scapulae (*p* = 0.048) activity levels, and decreased middle trapezius muscle stiffness (*p* = 0.014). Patients’ self-perception of change was rated favorably. **Conclusions**: After a scapular therapeutic exercise program, changes were observed in both self-reported and performance-based outcomes. These results need to be confirmed by a randomized controlled trial.

## 1. Introduction

Shoulder pain is the third most common musculoskeletal complaint [1], with a lifetime prevalence of 7 to 67% [2]. In 83% of cases, shoulder pain significantly impacts daily activities, job, and/or sport [3]. In these pain conditions, the relevance of the different shoulder complex joints, namely maintaining the function and integrity of the shoulder complex even if it is not possible to maintain its full mobility, has been highlighted [4]. Moreover, shoulder pain has been related to adaptations in local and surrounding structures [5], particularly with adaptations in scapular function.

The mentioned scapular adaptations, often manifested as changes in scapular kinematics, are commonly referred to as scapular dyskinesis [6] and/or as a reduction in the range of expected movements [7]. Additionally, scapular changes can be reflected by muscular alterations, which have mainly been reported as reduced activity in the lower trapezius (LT), middle trapezius (MT), and serratus anterior (SA) [8], as well as tightness or increased activity of the levator scapulae (LS) and pectoralis minor and tightness or changed activity of the upper trapezius (UT) [6]. Despite some uncertainty about the impact of these changes on shoulder pain, as they may not affect all patients and could be present in asymptomatic subjects [9], their association appears plausible. This is due to the role of the scapula, considering its positioning, motion, muscle-related activation, and length–tension balance [10,11], and consequently, the relevance of the scapulohumeral rhythm in providing proper shoulder alignment, stability, and motion [12]. Furthermore, the scapula is also important for allowing energy transference and connection between the upper limb and the trunk [3,13]. Moreover, previous studies have reported scapular adaptations as either a cause of shoulder pain [6] or a compensation that maintains shoulder and upper limb function and/or relieves pain in directly damaged structures [5]. However, these adaptations may also increase energy expenditure [13] or perpetuate/exacerbate shoulder pain [6]. In addition to the mentioned facts, other studies have also emphasized the relevance of directing and tailoring chronic shoulder pain rehabilitation programs based on scapular movement impairments [14], underscoring the need to assess scapular structures and presentation, including the possibility of dyskinesis. More recently, a study on rotator cuff diseases highlighted the implementation of personalized interventions that consider both variability and individual patient characteristics [15]. This approach may enhance the impact on the scapula–shoulder relationship, as well as the entire motor control rehabilitation.

From several rehabilitation possibilities, therapeutic exercise is often suggested as a primary-line intervention for shoulder pain [16,17]. This suggestion comes from its ability to induce hypoalgesia [18], induce neural adaptations (namely in muscle spindle or in the cortex) [19], and enhance motor control and function [16,17], as well as its possibility to be tailored to a patient’s specific needs. A recent study [19], which evaluated the combination of manual therapy and therapeutic exercises, also highlights the relevance of the intervention in promoting a better relationship between the action timings of scapula and glenohumeral muscles (possibly improving the scapulohumeral rhythm). Scapular-focused therapeutic exercises are particularly relevant for preventing shoulder pain perpetuation [20] and improving shoulder function [14] by restoring scapular positioning, motion (namely synchronous movement during shoulder motion), and muscular balance and length–tension [21] to the pre-injury state. These capabilities are particularly relevant for patients whose shoulder pain is associated with scapular dyskinesis, supporting the need to explore and test scapular exercise programs specifically designed for these subjects. Various exercises may be necessary to achieve these purposes, including muscular stretching [22], strengthening, and/or neuromotor control [3]. It is also important to note that, while scapular muscles collaborate in various scapular motions, they exhibit different activation timings [23] and moment arms throughout the movement [24]. Therefore, exercises should not target specific muscles exclusively but should, instead, emphasize their synergistic activity through global recruitment [13].

Despite the mentioned facts, there are still some conflicting findings regarding the effectiveness of scapular-focused therapeutic exercises in rehabilitating shoulder pain, whether specific or non-specific. In addition to the fact that its effect was often not studied in isolation from other techniques in the past, previous studies did not always establish a criterion for selecting exercises, nor did they consider the multifactorial aspects of chronic pain, neglecting the influence of parameters like psychosocial factors [25]. Moreover, while there are uncertainties about the contribution of some biomechanical parameters to improving shoulder pain and function, a limited number of studies have simultaneously assessed scapular motor control outcomes, such as kinematics and electromyographic activity level, allowing for the association of their findings [13]. Also, research about scapular therapeutic exercise effects on activities of daily living has been explored little in previous studies, which is a gap that needs to be filled, considering the high impact of chronic shoulder pain on upper limb functionality. So, continued investigation in this field could be useful to improve therapeutic exercise programs.

Considering the limitations mentioned, the present study intended to propose a program of scapular therapeutic exercises based on the level of muscular activity induced by each exercise and on a functional paradigm (involving distant structures and simulating activities of daily living). Then, it aimed to investigate the isolated short-term outcomes of an 8-week scapular therapeutic exercise program on several parameters relevant to subjects with chronic shoulder pain associated with scapular dyskinesis, firstly in self-reported outcomes and secondly in performance-based outcomes. Specifically, the present study assessed self-reported outcomes, such as shoulder pain and function, psychosocial factors, and self-impression of change, as well as performance-based outcomes like three-dimensional (3D) scapulothoracic motion, scapulohumeral rhythm and movement quality, scapular muscle electromyographic data (activity level), and muscle stiffness. Considering the mentioned facts, it was expected that the scapular therapeutic exercise program would positively impact several outcomes measured. These positive effects include restoring changes in scapular structures, alleviating symptomatology, and enhancing shoulder and upper limb function.

## 2. Materials and Methods

### 2.1. Study Design

An 8-week single-group pre- and post-intervention study was conducted, and two assessment moments were used to collect data on all outcomes, except for self-impression of change, which was only assessed after the intervention. At the baseline assessment moment (M0), before the scapular therapeutic exercise program began, anthropometric data and scapular clinical assessment test data were recorded. One week after the conclusion of the intervention program (9 weeks after the beginning of the intervention), each subject underwent reassessment (M1).

### 2.2. Participants

Subjects aged between 18 and 65 years old, experiencing chronic shoulder pain, were recruited from a higher education institution through an emailed questionnaire. Then, a researcher screened the eligibility of the volunteers. To participate in the present study, subjects needed to meet the following inclusion criteria: (a) experiencing shoulder pain (specifically in shoulder, deltoid, and/or scapular areas) lasting at least 3 months (duration of current episode or intermittent complaints over the last 3 months) and of moderate to high intensity [≥4 on numeric rating scale] at rest or during active shoulder movements, according to data reported by the participants; (b) presenting type I, II, and/or III in the scapular dyskinesis classification test, according to clinical tests performed by a physiotherapist. Participants presenting unilateral and bilateral shoulder pain were accepted, but only the most painful shoulder was considered in the case of bilateral pain.

Subjects were excluded if they had a history of shoulder fracture, dislocation, tears, infection, or neoplasm; had systemic, infectious, and/or neurological diseases; had undergone shoulder surgery at any time or received other treatment for shoulder symptoms in the last 3 months; presented cervical and/or thoracic pathologies; or experienced pain associated with active movements of these regions. Additionally, subjects were excluded if they also reported an inability to perform exercise, if they practiced competitive overhead sports or engaged in regular high-level upper extremity strength training, if they had a body mass index outside the range of 18.5–30 kg/m^2^; or if they had muscular skinfold measurements higher than 20 mm. No female participants were pregnant at the time of the study.

### 2.3. Outcome Measures

Participants were assessed on the following outcomes: (a) self-reported outcomes—shoulder pain and function, psychosocial factors, and self-impression of change [26]; (b) performance-based outcomes—3D kinematics of the scapula, scapulohumeral rhythm, and movement quality (including trunk lateral flexion, rotation or flexion/extension compensation, time-to-peak acceleration, and smoothness), as well as scapular muscular activity level [27] and scapular muscles stiffness [28].

### 2.4. Data Collection Protocol

Sociodemographic data and parameters related to eligibility criteria were obtained from the selection questionnaire.

Data collection took place in a human movement analysis laboratory (CIR—Center of Rehabilitation Research, from E2S, P.PORTO), from March to August 2023, with each researcher consistently executing the same tasks. To ensure an accurate positioning of participants and materials and to mark the intended shoulder range of motion, a universal goniometer (BASELINE^®^, Aurora, IL, USA) was used. For this tool, with a precision of 1°, an intraclass correlation coefficient (ICC) from 0.93 to 0.94 was observed [29,30].

Anthropometric measurements and scapular clinical assessment (including muscle length, scapular dyskinesis classification test, and scapular symptom modification tests) were performed before outcome assessment. Height (m) and body mass (kg) were measured using seca^®^ instruments (222 stadiometer and 760 scale, with 1 mm and 0.1 kg precision, respectively; seca^®^, Birmingham, UK).

The muscle length of the commonly tight muscles in shoulder pain was assessed following Appendix A (Table A1). A tape measure (Hoechstmass^®^, Sulzbach, Germany) was used for the pectoralis minor [31] and LS [32], while a manual assessment was used for the UT [33]. The scapular dyskinesis classification test and the scapular symptom modification tests (manual scapular assistance test and scapular repositioning test) were conducted based on prior recommendations [22,34] to characterize scapular positioning and potential scapular contribution to shoulder pain [3] (Appendix A, Table A1).

Shoulder pain intensity during activity was self-assessed using a 0–10 numeric rating scale, a validated and reliable tool with a minimal clinically important difference (MCID) of 2.17 for shoulder rehabilitation [35,36]. Shoulder function over the past week was self-assessed using the Shoulder Pain and Disability Index (SPADI) [0 (“no pain/no difficulty”, fully functional) to 100 (“worst pain imaginable/so difficult required help”)], validated for Portuguese population (ICC ≥ 0.89; MCID of 13.2) [37,38,39].

Scapular muscles stiffness was measured with MyotonPRO (Myoton AS., Tallinn, Estonia), a reliable digital palpation device [ICC = 0.775–0.971; standard error of measurement (SEM) = 7.76–43.23; minimal detectable change with a 95% confidence interval (MDC_95%_) = 21.51–119.84, for scapular muscles in chronic shoulder pain [28]]. Three measurements were obtained for the UT, MT, LT, upper/middle SA (SAup/mid), lower SA (SAlow), and LS muscles (Table 1), with participants seated and in isometric contraction (forearm in neutral position, elbow extended, and 90° of shoulder elevation in the scapular plane) [28]. A one-minute rest separated trials. Skinfold measurements at the same sites (averaged) were performed three times using the Harpender Skinfold Caliper (Baty International, England—precision of 0.2 mm).

Three-dimensional scapulothoracic motion, scapulohumeral rhythm, movement quality, and surface electromyography (EMG) were recorded during a functional drinking task comprising five phases: reaching, forward transport, drink, backward transport, and returning [47]. This task, representing a common daily activity and already used in other populations [47,48], involved lifting a 330 mL water bottle positioned at 90° of shoulder elevation in the scapular plane and at a distance similar to the length measured between the acromion and the trapezium–metacarpal joint of the upper limb being assessed [48]. The task was performed 5 times (with the average of the three central trials analyzed) at a self-selected speed, with a 30 s rest interval. Participants were seated, knees and hips at 90° flexion, feet flat on the floor, and the assessment started with the painful-side upper limb resting at the side of the body.

Kinematic data were collected at 100 Hz using the KINETIKOS CE-marked (Class I medical device) cloud-based platform (KINETIKOS, Coimbra, Portugal) and five inertial measurement units (MVN BIOMECH Awinda, Xsens Technologies, Enschede, the Netherlands) placed on the thorax, acromion, humerus, forearm, and back of the hand. Calibration and recordings followed the manufacturer’s recommendations and International Society of Biomechanics standards [49]. Scapular motion was analyzed in four degrees of freedom [abduction/adduction, elevation/depression, winging (the raising of the scapula’s medial border and inferior angle from the thorax), upward/downward rotation [50]], with shoulder (particularly, elevation) and trunk (lateral flexion, axial rotation, and forward flexion) movements also captured. The identification of the moments of the drinking task, data processing, and analysis were performed on the KINETIKOS platform. Outcome reliability from the present study is presented in Table 2.

Surface EMG was recorded for the trapezius, SA portions and LS using the wireless Trigno™ acquisition system (Delsys Inc., Natick, MA, USA), with six Trigno Avanti surface sensors (four-bar silver electrode, inter-electrode distance of 10 mm, 20–450 Hz, gain of 1000, common mode rejection coefficient > 80 dB, and sampling at 2000 Hz [51]). Following skin preparation (shaved, abraded, and cleaned with 70% isopropyl alcohol), sensors were positioned per Table 1 and affixed using an adhesive interface (Delsys, Inc.; Natick, MA, USA). EMGworks Acquisition and Analysis software (Delsys Inc., Natick, MA, USA) was used for signal quality, recording, filtering, normalization, and analysis. To synchronize kinematic and EMG data, a Trigno™ sensor on the hand collected accelerometry data and identified the beginning of movements. This synchronization allowed for an accurate alignment of movement phases between systems [27]. Reliability values for these outcomes are also presented in Table 2.

Given the multifactorial nature of chronic pain and the possible psychosocial influences on rehabilitation, two validated Portuguese questionnaires were applied: the Tampa Scale for Kinesiophobia [13–52 range, with higher scores indicating greater levels of perceived fear; ICC = 0.94, 0.98; minimal detectable change (MDC) = 4.16, which corresponds to 8% of the total score [52,53]] and the Pain Catastrophizing Scale (0–52 range, with higher scores representing greater levels of catastrophic thoughts; ICC = 0.773–0.823; MDC = 9.1 [54,55]).

At follow-up (M1), participants also rated their condition and perceived changes using the Portuguese version of the Patient Global Impression of Change Scale, ranging from 1 (no change or condition has worsened) to 7 (a great deal better and a considerable improvement that has made all the difference). The scale has strong construct validity (r = −0.822, in relation to a numeric rating scale) [56].

No participants reported pain during the assessment moments.

### 2.5. Intervention

All participants performed 24 sessions of scapular therapeutic exercises, each lasting between 25 and 40 min, conducted three times a week over an 8-week period. This duration was chosen to accommodate the anticipated changes, including initial neural adaptations and the following muscular structure adaptations [57]. A detailed description of the scapular therapeutic exercises included is presented below and in Appendix A (Table A2, Table A3 and Table A4). It is noteworthy that the exercise program was selected and personalized specifically for participants with shoulder pain associated with scapular dyskinesis, considering relevant scapular muscles for the different types of scapular dyskinesis. Adherence to and correct execution of the exercises were ensured through personalized supervision and feedback—either in person or via video call—provided by two physiotherapists with over 8 years of clinical experience. Additionally, the location where the exercises were performed was adjusted according to each participant’s needs and availability. Both physiotherapists had received training on the protocol.

The sessions were structured into three parts: (1) preparatory and warm-up exercises; (2) scapular neuromotor and strengthening exercises; (3) scapular and posterior capsule stretching exercises. Diaphragmatic breathing and core activation were used as preparatory exercises (Appendix A, Table A2) and were repeated 5 to 10 times. Then, shoulder circumduction exercises were used as a warm-up, with 15 repetitions forward and 15 backward (Appendix A, Table A2).

The main scapular neuromotor and strengthening exercises, aimed at enhancing scapular function and muscle synergistic activity, were selected from previous studies based on (a) the recruitment of at least 21% of the maximal or submaximal activity level to ensure moderate muscular recruitment [58] and neuromuscular retraining [59]; (b) the required shoulder range of motion; and (c) the number of scapular muscles recruited, at least to a moderate extent [58], prioritizing those commonly reported with reduced activity levels such as the LT, MT, and SA [8,24]. Considering these criteria, the rehabilitation program was divided into two phases, each including four main exercises: (a) the first four weeks (weeks 1 to 4)—exercises performed within a smaller shoulder range of motion for safer positioning; (b) the last four weeks (week 5 to 8)—exercises performed within a larger shoulder range of motion for increased demands [59]. Scapular multi-joint exercises (including trunk or lower limb movements) [59] were chosen whenever possible. Exercises were performed at a moderate pace in 2 sets of 10–15 repetitions with a 2 min rest period between sets and without causing the patient’s pain. During the first and fifth weeks, no resistance was applied. Red (medium resistance: 2nd, 3rd, 6th, 7th weeks) and green (heavy resistance: 4th and 8th) elastic bands (MVS In Motion, Belgium, Europe), with adjusted tension, were used progressively in subsequent weeks.

During the first four weeks, participants performed the “Lawnmower” [59,60], “Robbery” [59,60], “External rotation with squeeze” [60], and “External rotation at 0° of shoulder abduction, with scapular squeeze and trunk ipsilateral rotation” [61] exercises (Appendix A, Table A3). In the last four weeks, participants performed the “Diagonal of shoulder flexion-abduction-external rotation (D2F) [62]”, “Bilateral elevation with external rotation” [63], “External rotation at 90° of shoulder abduction, with scapular squeeze and trunk ipsilateral rotation” [61], and “Prone scapular plane abduction” [64] exercises (Appendix A, Table A3). In the fourth and eighth weeks, a daily activity task (sliding a box of 4 kg weight and an overhead height task with a full 0.5 L bottle [65], respectively) was added (Appendix A, Table A3).

At the end of each session, participants engaged in self-stretching exercises for the pectoralis minor [66], LS [67], UT [68], and posterior capsule (commonly tightened in scapular dyskinesis) [69]. Two repetitions of 15 s were performed in a static position (Appendix A, Table A4) until a sensation of tightness or minor discomfort was experienced.

### 2.6. Data Processing

EMG data were digitally filtered (2nd-order band-pass Butterworth filter: 20–450 Hz), and the root mean square was calculated using a sliding window of 100 samples. Acceleration data were low-pass-filtered (4th-order Butterworth filter: 4 Hz [70]) and synchronized with data from the KINETIKOS CE-marked (Class I medical device) cloud-based platform. Task onset was defined as the earliest instant at which hand acceleration exceeded the mean resting value by ±0.3 m/s^2^ [70]. Subsequently, drinking task phases were identified based on the following criteria [27,70]: (a) forward transport began at the point of maximum elbow extension, (b) the drink phase started with the initiation of shoulder elevation, (c) backward transport began at the shoulder’s maximum elevation, (d) the returning phase started with a new elbow’s maximum extension, and the ending was defined as the hand sensor’s acceleration returning within ±0.3 m/s^2^ of rest.

EMG signals were normalized using a submaximal isometric contraction, considering three 5 s repetitions for each test (Table 1), performed while holding a 1 kg dumbbell. A 30–60 s rest was adopted between trials.

The scapular rest position was calculated by averaging the first 5 s of static recording. Scapular and shoulder ranges of motion were determined by computing the angular difference between maximum and minimum joint positions. The scapulohumeral rhythm was calculated as the ratio between shoulder elevation/lowering and scapular upward/downward rotation. Movement quality was assessed by (a) trunk compensation, defined by peak variation of trunk movements; (b) percentage of time to peak acceleration, calculated during the reaching phase as the point of highest instantaneous hand acceleration [71], and normalized to task duration; (c) global movement smoothness [27], evaluated using the dimensionless jerk function derived from hand sensor data [72,73].

### 2.7. Statistical Analysis

The sample size was estimated using G*Power software 3.1 (Kiel University, Germany), based on the shoulder pain and function outcomes and their respective MCID values, to determine the number of participants needed to achieve clinically meaningful results. With a power of 0.8 and an alpha of 0.05, the parameters used were as follows: (a) for the Shoulder Pain and Disability Index [39], an MCID of 13.2 and a standard deviation (SD) of 20.71; (b) for the numeric rating scale [36], an MCID of 2.7 and an SD of 1.92. Considering both outcomes and an effect size of d = 0.63737, 17 participants were required to detect changes between pre- and post-intervention.

Data were analyzed using the Statistical Package for Social Sciences (IBM, Inc., New York, NY, USA), version 27, with a confidence level of 95%. Data normality was assessed using the Shapiro–Wilk test and visual inspection of the histogram.

To evaluate the changes throughout the exercise program, as appropriate, comparisons between pre- and post-intervention moments were conducted using a paired sample *t*-test or the Wilcoxon test. Data was presented as mean, SD, median, and interquartile range (IQR) or frequencies. Mean or median differences between groups for statistically significant results were presented based on changes in outcomes between M0 and M1. The effect size (Cohen’s d) was reported for significant results, except when a non-parametric test was used. Effect size values greater than 0.8 indicated a large effect, those around 0.5 indicated a moderate effect, and those less than 0.2 suggested a small effect [65].

To verify if the changes over the exercise program exceeded the instruments’ error for EMG and 3D kinematic outcomes, a reliability analysis was performed. Specifically, the intraclass correlation coefficient (ICC2, k) was calculated by a two-way mixed effects model with absolute agreement to determine the intra-rater reliability of the 3D scapulothoracic motion, scapulohumeral rhythm, movement quality, and scapular muscle electromyographic data. Reliability values were interpreted as follows: less than 0.15 indicates little reliability, 0.16–0.49 indicates low, 0.50–0.69 indicates moderate, 0.70–0.89 indicates high, and higher than 0.90 indicates very high reliability [74]. The standard error of measurement (SEM = SD × √1 − ICC) and the minimal detectable change (MDC_95%_ = SEM × √2 × 1.96) were also calculated.

## 3. Results

**Figure 1:**
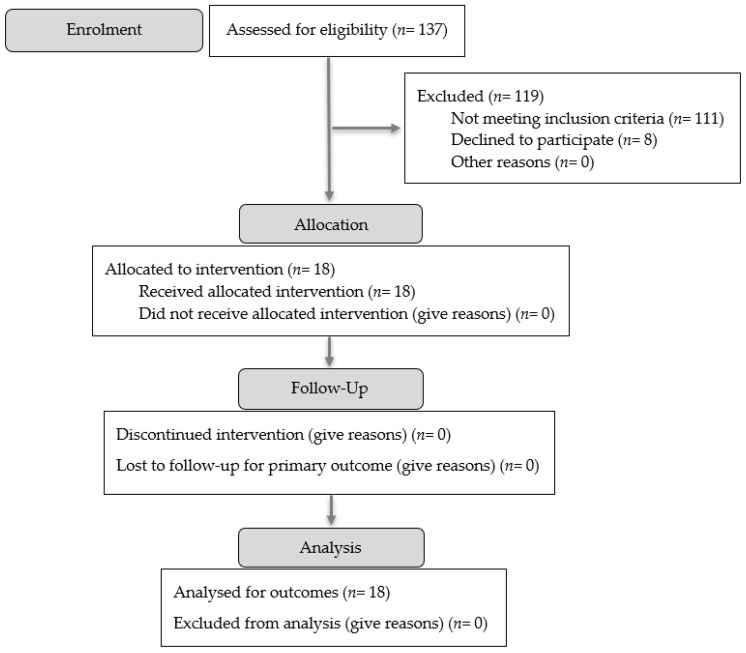
Modified CONSORT 2025 flow diagram for participants [75]. M0—baseline assessment moment; M1—assessment moment performed one week after the end of the scapular therapeutic exercise program.

A total of 137 answers were obtained in the selection questionnaire and assessed for eligibility. Among them, eighteen participants met the inclusion criteria and agreed to participate in the present study, while the remaining subjects were excluded for reasons outlined in Figure 1.

At baseline, the 18 participants in the present study were characterized by the outcomes presented in Table 3.

No adverse effects associated with the intervention protocol were reported by any of the participants. Except for one participant who completed one fewer session, all participants completed the proposed 24 sessions.

### 3.1. Self-Reported Outcomes: Shoulder Pain and Function, Psychosocial Parameters, and Self-Impression of Change

At M1, a significant reduction in shoulder pain (*p* < 0.0001) and pain catastrophizing (*p* = 0.004) and an increase in shoulder function (*p* < 0.0001) were observed. The increase in shoulder function was supported by a large effect size. Specifically, there was a median difference of 4.5 points on the numeric rating scale, a median difference of 8.5 points on the Pain Catastrophizing Scale, and a mean difference of 23.55 points in the Shoulder Pain and Disability Index (Table 4). Participants rated their condition and changes on the Patient Global Impression of Change Scale from 4 to 7. Specifically, the conditions/changes were rated as 4 (somewhat better, but the change has not made any real difference) by one participant, 5 (moderately better, and a slight but noticeable change) by four participants, 6 (better and a definite improvement that has made a real and worthwhile difference) by five participants, and 7 by eight participants.

### 3.2. Performance-Based Outcomes: Scapular Kinematics, Electromyographic, and Muscle Stiffness Outcomes

**Figure 2:**
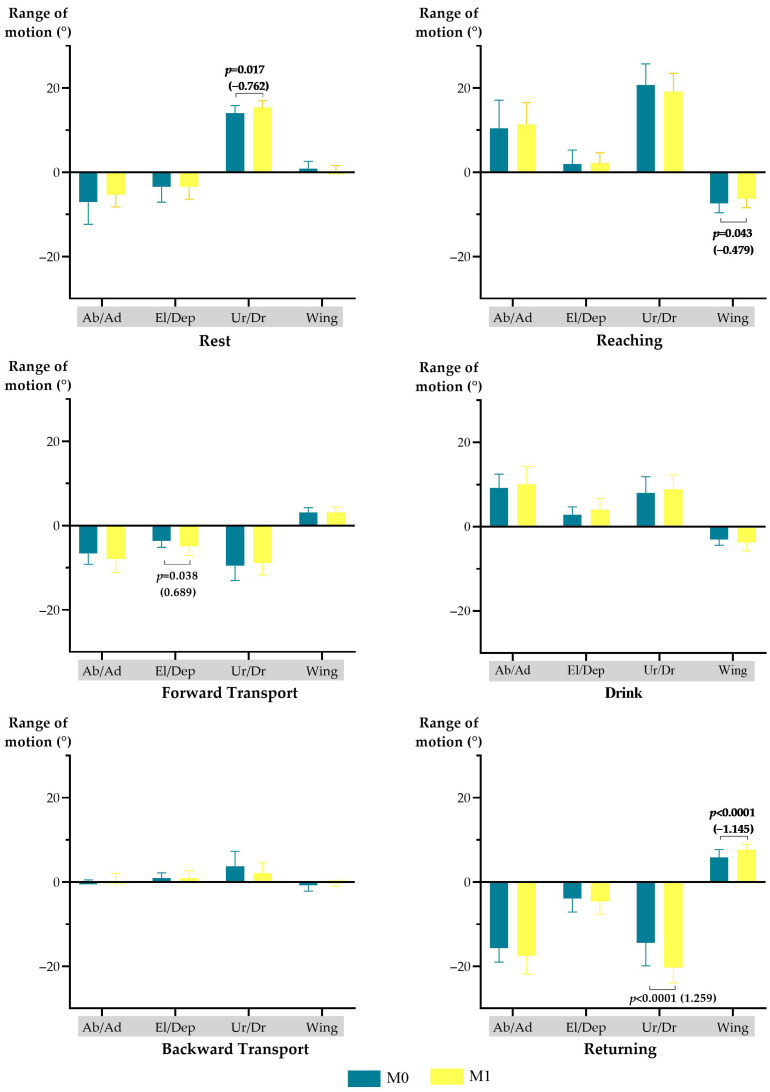
Scapulothoracic rest positioning and motion, during the drinking task, at M0 and M1. Ab/Ad—abduction (+) or adduction (−); El/Dep—elevation (+) or depression (−); STh motion—scapulothoracic motion; Ur/Dr—upward rotation (+) or downward rotation (−); Wing—winging (+). Only significant results, represented by the *p*-value and, in parentheses, by effect size, are presented in the figure.

Considering the 3D scapulothoracic motion, scapulohumeral rhythm, and movement quality data, after the intervention (M1), several changes were observed through comparison with the baseline assessment (M0). Particularly, at M1, the following observations were made: (a) at rest, an increase in scapular upward rotation (mean difference of 1.312°; *p* = 0.017, moderate effect size); (b) during the reaching phase, a reduction in scapular winging (mean difference of 1.056°; *p* = 0.043, small effect size); (c) during the forward transport phase, an increased range of scapular depression (mean difference of 1.292°; *p* = 0.038, moderate effect size); (d) during the return phase, an increased range of scapular downward rotation (mean difference of 5.884°; *p* < 0.0001, large effect size) and scapular winging (mean difference of 1.852°; *p* < 0.0001, large effect size) (Figure 2); (e) a decreased global movement smoothness (median difference of 0.777; *p* = 0.003) (Table 5).

**Figure 3:**
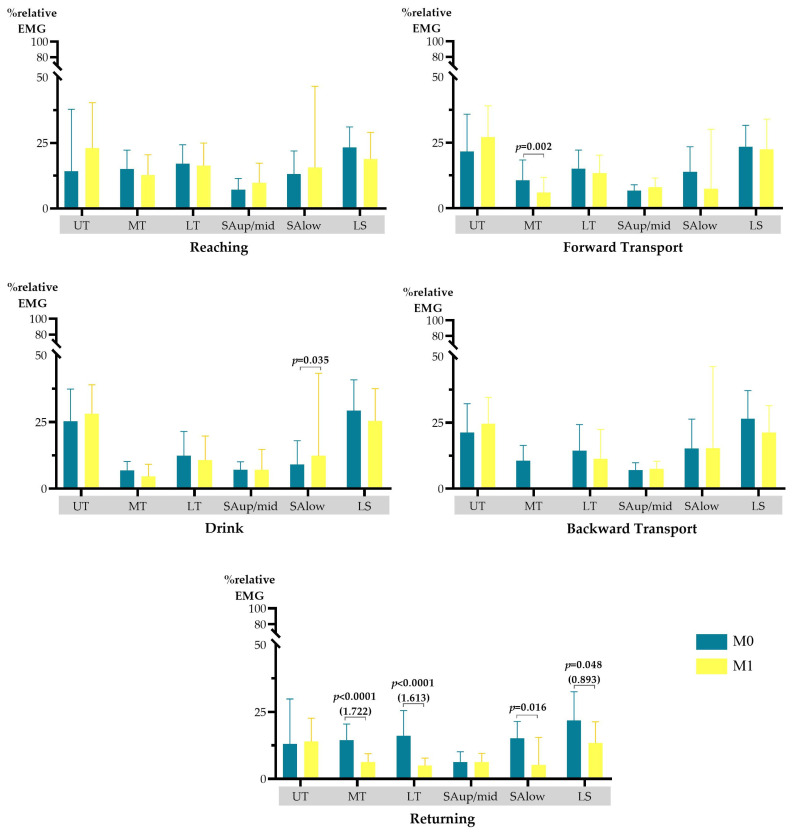
Scapular muscular activity level, during the drinking task, at M0 and M1. LS—levator scapulae; LT—lower trapezius; MT—middle trapezius; SAlow—serratus anterior lower portion; SAup/mid—serratus anterior upper/middle portion; UT—upper trapezius. Only significant results (and respective effect sizes) are presented in the figure.

Considering the scapular muscular activity, at M1, a statistically significant reduction in the MT’s muscular activity level was observed during forward transport (median difference of 4.576%, *p* = 0.002) and return phases (mean difference of 8.206%, *p* < 0.0001, large effect size). Additionally, a significant reduction in LT (mean difference of 11.187%; *p* < 0.0001, large effect size), SAlow (median difference of 9.893%; *p* = 0.016), and LS (mean difference of 8.388%; *p* = 0.048, large effect size) muscular activity was observed during the return phase. A significant increase in SAlow muscular activity during the drink phase (median difference of 3.289; *p* = 0.035) was observed (Figure 3).

Furthermore, a reduction in MT muscle stiffness, with a mean difference of 57.39 N/m (*p* = 0.014, supported by a moderate effect size), was observed (Table 4).

## 4. Discussion

This study, with a pre–post-intervention design, aimed to investigate self-reported and performance-based outcomes before and after a scapular therapeutic exercise program. The exercise program was applied exclusively to patients with chronic shoulder pain associated with scapular motor control impairments—more specifically, scapular dyskinesis—to optimize rehabilitation outcomes. The exercise dose was adjusted according to rehabilitation goals, supervision and feedback were personalized throughout the entire exercise program, resistance levels were adjusted to each participant’s requirements, and the intervention setting was tailored to the participants’ personal context and availability. Furthermore, both the task evaluated during assessment moments and the inclusion of exercises mimicking activities of daily living were chosen to support the ultimate rehabilitation goal—transferring learned motor skills to each patient’s everyday activities—thus preparing them for a full functional return, aligned with their personal realities and daily needs. One week after the completion of the exercise program, several changes were observed—including in pain intensity and catastrophizing, shoulder function, kinematics and electromyographic outcomes, and muscle stiffness—as discussed below.

### 4.1. Self-Reported Outcomes

At baseline, participants reported moderate pain intensity [76] and a low impact on shoulder function [77]. Although this initial characterization did not suggest severe impairment, significant changes were observed in these outcomes, exceeding the MCID reported in previous studies [36,39]. These findings are consistent with prior research on the effects of scapular-focused exercises [78,79] and suggest greater changes than those reported without intervention [80]. However, due to the design of the present study, definitive conclusions about the effectiveness of the intervention cannot be drawn, and these findings must be confirmed through a randomized controlled trial study. Our findings regarding pain and function align with participants’ self-impression of change, as most participants considered that the scapular exercise program led to significant changes. Specifically, 14 participants described the changes as useful and considerable. These results are consistent with previous studies investigating scapular-focused interventions for chronic shoulder pain [81] or subacromial impingement syndrome [82].

At baseline, participants also demonstrated a relatively favorable psychosocial profile, with no pain catastrophizing [83] and low levels of kinesiophobia [84]. Despite the statistically significant reduction in pain catastrophizing observed at M1, similar to what has been reported in previous studies [78,81], the median difference observed was less than the MDC for the Pain Catastrophizing Scale [85].

### 4.2. Scapular Kinematics, Electromyographic, and Muscle Stiffness Outcomes

An increased upward rotation was observed during the rest period preceding the drinking task; however, this change was lower than the MDC_95%_ values presented in the Materials and Methods section and thus could not imply clinical relevance. During the task itself, an increased range of scapular motion at M1 was observed, consistent with previous studies on rehabilitation programs [69,81]. Despite the limitations of the study design, this finding seems relevant, considering existing evidence of reduced scapular motion in shoulder pain conditions [7,12,65] and the low likelihood of spontaneous recovery [86]. Therefore, future studies should test the hypothesis that the proposed intervention program may enhance upper limb function and/or improve the control of scapular dyskinesis. However, not all assessed movements exhibited changes in scapular kinematics, which is consistent with prior research. Unexpectedly, global movement smoothness decreased at M1, exceeding the MDC_95%_. However, this change has not been previously reported in shoulder pain patients compared to asymptomatic subjects, and the observed value remained close to that of asymptomatic controls [27]. Moreover, a recent study on individuals with recurrent shoulder instability—both operated and non-operated—suggested that these patients exhibited movement patterns more aligned with the theoretical model of the greatest smoothness than healthy controls. The authors proposed that this could be due to the adoption of more stereotyped and pre-planned motor strategies with reduced reliance on feedback-based adjustments, potentially as a protective mechanism to minimize pain or reinjury [87]. While increased movement smoothness is often interpreted as a sign of motor efficiency, in this context, it may reflect a compensatory strategy rather than improved motor control. However, decreased smoothness observed after therapeutic exercises may also result from fatigue, high exercise volume, or repetitive loading. These factors were mitigated in the present study by alternating training days, the use of recommended exercise dose (in terms of number of exercises, sets, and repetitions), and a progressively structured rehabilitation protocol.

Increased activity of the SAlow was observed during the drink phase, although it did not exceed the MDC_95%_. This finding may anticipate a potential increase in the participation of the SA in scapular motion and stabilization, which could be particularly relevant given its commonly reported reduced activity in subjects with shoulder pain [8,24,27] and considering its susceptibility to weakness. Nevertheless, future studies are needed to determine whether this change is attributable to natural recovery or due to intervention itself. In turn, a reduction in muscle activity was observed in the MT (during the forward transport and returning phases), LT, SAlow, and LS (during the returning phase). If confirmed by a future randomized controlled trial, these changes may be clinically relevant, as a previous study has reported increased activity in these muscles compared to asymptomatic subjects [27].

The significant reduction observed in muscle stiffness of the MT aligns with previous findings that reported differences in this outcome between participants with (dominant or non-dominant) and without chronic shoulder pain [28]. This result is also consistent with studies assessing the effect of several interventions, particularly in the UT [1,43]. Although this change did not exceed the previously calculated MDC_95%_ [28], thus being unable to guarantee clinical relevance, it highlights the need for future studies to confirm the impact of the scapular therapeutic exercise program on this outcome.

Despite the limitations of the present study design, the several changes observed following the intervention may be related to the specific eligibility criteria used for sample selection [88,89] as well as to the implementation of scapular therapeutic exercises specifically tailored to the presence of scapular dyskinesis in participants with chronic shoulder pain. The exercises performed in the present study were previously identified as effective for addressing scapular impairments associated with shoulder pain [14,20], as they target scapular muscles commonly affected in this population [6,22,60,61,62,63,64].

### 4.3. Limitations

The present study had a few limitations. Firstly, the use of a single-group design limits both the internal validity and the generalizability of the results. This design does not guarantee that the observed changes were not influenced by factors such as the natural history of shoulder pain or subjectivity bias from participants and researchers, which may have led to an overestimation of the changes observed in the assessed outcomes. Therefore, given these limitations, future studies should consider these results as preliminary data for a possible randomized controlled trial aimed at evaluating the effectiveness of the proposed scapular therapeutic exercise program. Second, the lack of information on the pectoralis minor could have limited the interpretation of the results, as alterations in this muscle are common in subjects with shoulder pain conditions [6,14,22] and may affect scapular motion [22]. Although its length was assessed at baseline and no shortening was identified, this data was only used for characterization. Therefore, future studies should include an assessment of pectoralis minor muscular activity. Third, due to practical challenges in implementing the experimental protocol, the level of physical activity performed beyond the exercise program was not controlled. Fourth, although each variable was consistently collected or analyzed by the same researcher, some variables demonstrated low reliability values. Fifth, to reduce measurement errors and soft tissue artifacts, scapular kinematics were only assessed up to 120° of the shoulder range of motion. Finally, although the present study aimed to include outcomes recommended by the Initiative on Methods, Measurement, and Pain Assessment in Clinical Trials (IMMPACT) [26], as well as clinically relevant outcomes for healthcare professionals involved in the rehabilitation of chronic shoulder pain associated with scapular dyskinesia, the large number of variables assessed and the multiple comparisons performed may have increased the risk of false-positive results. Although efforts were made to minimize this risk—such as reporting the clinically relevant results or those exceeding the minimal detectable change—the findings of the present study should be considered as hypothesis-generating and confirmed in future studies with stronger methodological designs.

## 5. Conclusions

After a scapular therapeutic exercise program tailored for participants with chronic shoulder pain associated with scapular dyskinesis, a reduction in shoulder pain and pain catastrophizing was observed, along with improvements in shoulder function. A decrease in scapular muscle activity, middle trapezius muscle stiffness, and global movement smoothness was also noted, while scapular positioning and range of motion increased. Most participants reported favorable changes in their condition. However, not all findings reached clinical relevance or exceeded the minimal detectable change, which, combined with the absence of a control group, limits the strength of the conclusions. Therefore, all the results should be interpreted with caution, as they are derived from a single-group study.

## Figures and Tables

**Table 1:** Description of the assessment locations for scapular muscle stiffness and surface electromyography.

Muscle	Muscle Stiffness Assessment Location	EMG Electrodes’ Location	SVIC Positioning
UT	Midpoint between the 7th cervical spinous process and the acromion angle [40].	2 cm laterally to the middle of the line connecting the 7th cervical spinous process and the posterior tip of the acromion [41].	Seated, with shoulder at 90° abduction and neck in same-side inclination, opposite-side rotation and extension [42].
MT	Midpoint between the 4th thoracic spinous process and the medial border of the scapular spine [43].	Midpoint between the scapular spine’s root and the 3rd thoracic spinous process [8].	Prone, with shoulder in horizontal abduction and lateral rotation [42].
LT	Midpoint between the 6th thoracic spinous process and the medial border of the scapular spine [43].	At 2/3 of the line connecting the scapular spine’s root and the 8th thoracic spinous process [41].	Prone, with shoulder in abduction (obliquely at 135°) [42].
SAup/mid	Over the 4th rib, in the middle of the latissimus dorsi and the pectoralis major [44].		Supine, with shoulder in flexion, adduction, and lateral rotation [44].
SAlow	Over the 7th rib, in the middle of the axilla [44].		Supine, with shoulder at 125° of forward flexion [44].
LS	Midpoint between the sternocleidomastoid and the UT, at C4/C5 level [45].		Prone, with shoulder in horizontal abduction and lateral rotation [46].

Legend: EMG—electromyography; LS—levator scapulae; LT—lower trapezius; MT—middle trapezius; SAlow—serratus anterior lower portion; SAup/mid—serratus anterior upper/middle portion; SVIC—submaximal voluntary isometric contraction; UT—upper trapezius. The SAup/mid and SAlow position test was adjusted from seated to supine position, ensuring two points of resistance as in the original test [44].

**Table 2:** Intra-rater test–retest reliability for 3D scapulothoracic motion, scapulohumeral rhythm, movement quality, and scapular muscle electromyographic data.

		ICC	95% CI	SEM	MDC_95%_
EMG data	UT (%)	0.78	[0.16; 0.94]	3.83	10.60
	MT (%)	0.56	[−1.48; 0.92]	1.39	3.86
	LT (%)	0.69	[−0.06; 0.92]	3.64	10.09
	SAup/mid (%)	0.42	[6.15; 0.97]	4.14	11.46
	SAlow (%)	0.89	[0.55; 0.98]	2.33	6.46
	LS (%)	0.93	[0.38; 0.99]	0.75	2.08
3D kinematic data	Scapular Ab/Ad (°)	0.14	[−2.97; 0.78]	1.41	3.91
	Scapular El/Dep (°)	0.73	[0.01; 0.92]	0.36	0.99
	Scapular Ur/Dr (°)	0.63	[−0.19; 0.90]	0.35	0.98
	Scapular Winging (°)	0.66	[−0.09; 0.90]	0.23	0.63
	Scapulohumeral Rhythm	0.38	[−1.30; 0.83]	0.40	1.11
Movement quality data	Trunk Fw. Flexion (°)	0.71	[−0.21; 0.93]	1.11	3.07
	Trunk Ax. Rotation (°)	0.35	[−3.14; 0.87]	2.74	7.60
	Trunk Lat. Flexion (°)	0.74	[−0.15; 0.94]	0.65	1.81
	% Time to Peak Acceleration	0.63	[−0.50; 0.92]	5.53	15.33
	Global Movement Smoothness	0.76	[−0.03; 0.94]	0.11	0.30

Legend: Ab/Ad—abduction (+) or adduction (−); CI—confidence interval; El/Dep—elevation (+) or depression (−); EMG—electromyographic; ICC—intraclass correlation coefficient; LS—levator scapulae; LT—lower trapezius; MT—middle trapezius; MDC_95%_—minimal detectable change with a 95% CI; SAlow—serratus anterior lower portion; SAup/mid—serratus anterior upper/middle portion; SEM—standard error of measurement; Trunk Ax. Rotation—trunk axial rotation [(+), to the side of the assessed upper limb; (−), to the opposite side of the assessed upper limb]; Trunk Fw. Flexion—trunk forward flexion (−), or extension (+); Trunk Lat. Flexion—trunk lateral flexion [(+), to the side of the assessed upper limb; (−), to the opposite side of the assessed upper limb]; Ur/Dr—upward rotation (+) or downward rotation (−); UT—upper trapezius.

**Table 3:** Sample demographic characteristics.

Sample (*n* = 18)			
Height (m)		(mean ± SD)	1.66 ± 0.06
Body mass (kg)			63.50 ± 8.35
Body mass index (kg/m^2^)			23.19 ± 2.94
Age (years)			43.44 ± 13.93
Length of pectoralis minor (cm)			9.35 ± 0.69
Length of levator scapulae (cm)			8.89 ± 0.76
Sex	Female	[frequency (*n*)]	89% (16)
	Male		11% (2)
Upper limb dominance	Right-handed		89% (16)
	Left-handed		11% (2)
Shoulder pain location	Dominant side		44% (8)
	Non-dominant side		56% (10)
Scapular dyskinesis type	Type II		39% (*n* = 7)
	Type III		17% (*n* = 3)
	Type II + III		44% (*n* = 8)
Scapular modification tests	Negative tests		61% (*n* = 11)
	Positive scapular assistance test		17% (*n* = 3)
	Positive scapular reposition test		11% (*n* = 2)
	Positive scapular assistance test and scapular reposition test		11% (*n* = 2)

Legend: SD—standard deviation.

**Table 4:** Comparison of shoulder pain, shoulder function, psychosocial, and muscle stiffness data between M0 and M1.

Outcome Domain	Outcome		Assessment Moment’s		Comparison Between M0 and M1		
			M0	M1	t/U	*p*	Effect Size
**Shoulder function**	Shoulder Pain and Disability Index (SPADI)	(mean ± SD)	31.72 ± 17.68	8.17 ± 6.18	6.091	<0.0001 *	1.44
**Pain**	Numeric rating scale	(median ± IQR)	5.50 ± 3.00	1.00 ± 2.00	0.000	<0.0001 *	-
**Psychosocial factors**	Pain Catastrophizing Scale		10.00 ± 9.75	1.50 ± 9.00	9.500	0.004 *	-
	Tampa Scale for Kinesiophobia		24.00 ± 6.00	20.50 ± 12.50	42.500	0.106	-
**Muscle stiffness (N/m)**	MT stiffness	(mean ± SD)	452.48 ± 110.91	395.09 ± 80.02	2.732	0.014 *	0.64
	UT stiffness		388.00 ± 83.89	390.98 ± 67.72	−0.215	0.833	−0.05
	LT stiffness		516.63 ± 127.41	504.00 ± 97.45	0.747	0.465	0.18
	LS stiffness	(median ± IQR)	223.17 ± 57.42	249.59 ± 74.00	113.000	0.231	-
	SAup/mid stiffness		179.50 ± 43.33	187.83 ± 8067	110.500	0.276	-
	SAlow stiffness		182.83 ± 98.08	175.67 ± 95.33	83.000	0.758	-

Legend: IQR—interquartile range; LS—levator scapulae; LT—lower trapezius; M0—baseline assessment moment; M1—last assessment moment; MT—middle trapezius; SD—standard deviation; SAlow—serratus anterior lower portion; SAup/mid—serratus anterior upper/middle portion; UT—upper trapezius. Significant results are indicated with an asterisk (*).

**Table 5:** Comparison of scapulohumeral rhythm and movement quality data between M0 and M1.

			Rest Before Drinking Task			Entire Drinking Task		
			(mean ± SD)	S-Value (t)/*p*-Value	Effect Size	(mean ± SD)	S-Value (t)/*p*-Value	Effect Size
Scapulohumeral rhythm		M0				1.577 ± 0.51	0.315/0.757	0.072
		M1				1.545 ± 0.37		
Movement Quality	Global movement smoothness	M0				2.164 ± 0.86	−3.567/0.003 *	−0.865
		M1				2.941 ± 0.23		
	Time to peak acceleration (%)	M0				54.055 ± 15.71	0.191/0.851	0.045
		M1				53.284 ± 10.26		
	Trunk Fw. Flexion (°)	M0	0.380 ± 0.44	1.228/0.240	0.317	−6.847 ± 3.86	1.431/0.174	0.369
		M1	0.134 ± 0.54			−8.407 ± 3.96		
						(median ± IQR)	S-value (U)/*p*-value	
	Trunk Lat. Flexion (°)	M0	0.001 ± 0.42	0.139/0.891	0.036	−0.138 ± 2.78	82.000/0.211	
		M1	−0.020 ± 0.31			1.454 ± 5.02		
			(median ± IQR)	S-value (U)/*p*-value				
	Trunk Ax. Rotation (°)	M0	0.003 ± 0.51	59.000/0.407		5.257 ± 4.92	59.000/0.683	
		M1	−0.015 ± 0.37			5.125 ± 9.03		

Legend: IQR—interquartile range; M0—baseline assessment moment; M1—last assessment moment; SD—standard deviation; S-value (statistical value); Trunk Ax. Rotation—trunk axial rotation [(+), to the side of the assessed upper limb; (−), to the opposite side of the assessed upper limb]; Trunk Fw. Flexion—trunk forward flexion (−), or extension (+); Trunk Lat. Flexion—trunk lateral flexion [(+), to the side of the assessed upper limb; (−), to the opposite side of the assessed upper limb]. Significant results are indicated with an asterisk (*).

## Data Availability

The datasets presented in this article are not readily available because the data are part of a major study. Requests to access the datasets should be directed to the first author or to the corresponding author.

## Abbreviations

The following abbreviations are used in this manuscript:

3D	Three-dimensional
Ab/Ad	Abduction/adduction
CI	Confidence interval
El/Dep	Elevation/depression
EMG	Electromyography
ICC	Intraclass correlation coefficient
IQR	Interquartile range
LT	Lower trapezius
LS	Levator scapulae
M0	Baseline assessment moment
M1	Assessment moment one week after the conclusion of the intervention
MCID	Minimal clinically important difference
MDC	Minimal detectable change
MDC_95%_	Minimal detectable change with a 95% confidence interval
MT	Middle trapezius
SA	Serratus anterior
SAlow	Serratus anterior lower portion
SAup/mid	Serratus anterior upper/middle portion
SD	Standard deviation
SEM	Standard error of measurement
STh motion	Scapulothoracic motion
S-value	Statistical value
Trunk Ax. Rotation	Trunk axial rotation
Trunk Fw. Flexion	Trunk forward flexion
Trunk Lat. Flexion	Trunk lateral flexion
UT	Upper trapezius
Ur/Dr	Upward rotation/downward rotation

## Appendix A

**Table A1:** Scapular clinical assessment tests (steps and intra-rater reliability).

Scapular Test		Tests’ Steps	Results	Intra-Rater Reliability
Pectoralis minor length measurement		- Participant standing and relaxed; - Measurement of the distance (cm) between the caudal edge of the 4th rib, at the sternum, and the inferomedial aspect of the coracoid process [31,66], at the end of expiration; - Normalization of the obtained value by the participant’s height (cm) and multiplication by 100.	Short: values < 7.44 [90]	Intra-rater ICC from 0.82 to 0.95 [91,92]
LS length measurement		- Participant standing, with arms relaxed and the head and cervical in neutral position; - Measurement of the distance between the 2nd cervical transverse process and the superior medial angle of the scapula [32], at the end of expiration; - Normalization of the obtained value by the participant’s height (cm) and multiplication by 100.	Short: values ≤ 6.9 [93]	Intra-rater ICC from 0.94 to 0.98 [32,94]
Upper trapezius length		- Participant seated with feet on the floor and in neutral posture; - Passive contralateral lateral flexion and ipsilateral rotation of the neck until UT maximum length; - Same-side shoulder elevation followed by another passive neck movement [33].	Short: further motion after elevating the same-side shoulder [33]	Intra-rater test–retest agreement of κ = 0.72 [33] (data only available for asymptomatic subjects)
Scapular dyskinesis classification test		- Participant standing with neutral posture; - Scapular assessment (through observation), at rest and during 3 to 5 repetitions of bilateral upper limb elevation and lowering in the frontal plane [3,95].	- Type I: prominent scapula inferior angle [31]; - Type II: prominent scapular medial border [31]; - Type III: excessive scapular elevation and/or excessive/insufficient scapular upward rotation [31]; - Type IV: balanced position/movement of both scapulae [31].	Intra-rater reliability of κ = 0.49–0.59 [95]
Scapular symptom modification tests	Scapular assistance test	- Participant standing; - Perform shoulder elevation without assistance for comparison with the same movement performed with assistance for scapular motions, namely by pushing the scapular inferior medial border upward and laterally and stabilizing the upper medial border [34].	Positive: symptoms diminished/relieved [96]	Intra-rater ICC from 0.98 to 0.99 [97]
	Scapular reposition test	- Participant standing; - Isometric maintenance of shoulder elevation against resistance for comparison with the same task performed with assistance in scapula’s medial border stabilization, to a more retracted position on the thorax, and posterior tilt [34].	Positive: pain reduction or increased shoulder elevation strength [34]	Intra-rater reliability of κ = 0.43 [98]

Legend: ICC—intraclass correlation coefficient; LS—levator scapulae.

**Table A2:** Preparatory and warm-up exercises.

Exercise					
Type	Name	Position			Description
Preparatory exercise	Diaphragmatic breathing				Initial position: - Seated or standing upright; - Place one hand on your chest and the other on your belly. Movement: - Feel the movement in your hands while breathing: the hand on your chest should feel minimal movement, while the hand on your belly should be the one moving the most. This hand should move forward during inhalation and backward during exhalation.
	Core activation		→		Initial position: - Seated or standing upright. Movement: - While breathing, activate/contract your abdominal muscles.
Warm-up exercise	Shoulder circumduction		→		Initial position: - Seated or standing upright; - Maintain both arms extended along your body. Movement: - While maintaining diaphragmatic breathing and core activation, gently rotate your shoulders forward and then backward.

Footnote: Preparatory exercises were selected to teach correct diaphragmatic breathing patterns; promote improved posture; enhance periscapular muscle activation, stabilization, and energy transfer from other body segments; and reduce injury of the peripheral joints through activation of the abdominal muscles. In turn, warm-up exercises were included to help increase local temperature and mobility and, consequently, improve the performance of the remaining exercises.

**Table A3:** Scapular neuromotor and strengthening exercises.

Interv. Phase		Exercise				
	Name	Initial Position Without Resistance	Final Position Without Resistance	Initial Position with Resistance	Final Position with Resistance	Muscles Worked *
1st	Lawnmower	- Stand upright - Knees semi-flexed - Trunk flexed forward and rotated to the contralateral side of the painful shoulder - Hand from the side of the painful shoulder, at the level of the contralateral patella	- Lower limbs and trunk extension until reaching a vertical position - Trunk rotation to the side of the painful shoulder - Elbow from the side of the painful shoulder, moved until the waist level in the direction of the back pocket - Retraction of both scapulae	- Repeat the exercise steps without resistance, but hold a band between both hands at a distance equal to shoulder width apart - The hand of the pain-free limb (or the less painful one) will serve as a fixed point for the band and must be kept near the thigh on the same side as the pain-free limb	Movement: - Repeat the exercise steps without resistance - Move the elbow on the side of the pain up to waist level and pull it back as if reaching for the back pocket, stretching the band	- LT [59] - SA and UT [59,60]
	Robbery	- Stand upright - Lean your trunk slightly forward while bending the knees slightly - Use both upper limbs in this exercise - Keep the elbows extended/straightened - Place the palms of both hands facing towards the legs [59,60]	- Extend both lower limbs and the trunk until reaching a vertical position - Simultaneously, bring both elbows towards the trunk, bending them at a 90° angle towards the back pocket. Rotate the arms so that your hands face outward and away from each other - Squeeze (retract and lower) both scapulae	- Repeat the exercise steps described without resistance - Additionally, each hand should hold a part of the band, keeping them apart at shoulder width - The elbows should remain extended, and the palms of both hands should face the legs	- Extend both lower limbs and the trunk until reaching a vertical position - Simultaneously, bring both elbows towards the trunk, bending them at 90° towards the back pocket. Rotate the arms so that your hands face outward and away from each other - Squeeze (retract and lower) both scapulae	- MT [60] - LT [59] - SA and UT [59,60]
1st	Shoulder external rotation with squeeze	- Stand upright - The elbow on the side with the shoulder pain (or more painful) should be flexed/bent at a 90° and kept close to the body	- Rotate the hip on the side with shoulder pain (or more painful) outward, in the direction of the limb performing the exercise - Simultaneously, rotate the arm on the side with shoulder pain (more painful) outward, keeping the elbow close to the trunk, so that the hand is turned outward and away from the body’s midline, bringing it towards the back pocket - Squeeze both scapulae for 3 s	- Repeat the steps of the exercise without resistance - Additionally, hold the band with both hands, which should be shoulder-width apart, bending the elbows to form a 90° angle and being careful to keep them close to the body - The hand of the limb that is not performing the exercise will serve only as a fixed point for the band	- Rotate the hip on the side of the shoulder with pain (or more painful) outwards in the movement’s direction - Simultaneously, bring your hand on the side of the shoulder with pain (or more painful) in that direction, always keeping the elbow close to the body and towards the back pocket, pulling the band - Squeeze both scapulae for 3 s	MT, LT, and SA [60]
	Shoulder external rotation at 0° with scapular squeeze and trunk ipsilateral rotation	- Sit with your knees bent at 90° and feet resting on the ground. Look straight ahead and keep the trunk and back straight, in a neutral position, aided by abdominal contraction - The elbow on the side with shoulder pain (or more painful) should be flexed/bent at 90° and kept close to the body - The hand of the limb experiencing pain should be turned in the direction of the opposite knee	- Perform trunk rotation to the side of the painful shoulder - Simultaneously, rotate your hand on the side of the shoulder with pain (or more painful) outward to the maximum extent while keeping the elbow close to the trunk - Squeeze both scapulae for 3 s	- Repeat the steps of the exercise without resistance - Additionally, hold the band with both hands shoulder-width apart, with the elbows bent at 90°, and be careful to keep them close to the body - The hand of the limb not performing the exercise will serve only as a fixed point for the band and can rest by your thigh - The hand on the side with shoulder pain should be turned towards the opposite knee	- Rotate the trunk towards the side of the shoulder with pain (more painful) - Simultaneously, bring the hand on the side of the shoulder with pain (or more painful) in that direction as well (as much as possible), pulling the band and keeping the elbow close to the body - Squeeze both scapulae for 3 s	LT and SA [61]
1st	Sliding a box task	-	-	- Sit in front of a table with knees bent at 90° and feet resting on the floor. - Maintain a straight cervical, trunk, and pelvis alignment - The height of the table should not exceed the level of your chest - Additionally, place a box on the table containing a total weight of 4 kg [65]	- With the hand on the side with shoulder pain (or the side experiencing more pain), push the box along the table	-
2nd	Diagonal flex-abd-ext.rot (D2F)	- Stand upright, looking straight ahead and maintaining a neutral position of the trunk and pelvis, with the assistance of abdominal contraction (engaged/activated core) - Position the hand of the upper limb with shoulder pain (or more painful) at the level of the hip of the opposite lower limb, and rotate it inward toward the hip	- Elevate the upper limb with shoulder pain (or more painful), opening it outward and raising it as high as possible. - Simultaneously, rotate the hand on the side of the pain (or more painful) outward	- Repeat the steps of the exercise without resistance - Additionally, the hand of the pain-free limb (or the limb with less pain) should hold a portion of the band near the thigh on its side, serving as a fixed point for the band. - The hand of the upper limb with shoulder pain (or more painful) should hold the other end of the band near the thigh of the opposite lower limb. Ensure that the length of the band matches the width of your shoulders	- Elevate the upper limb with shoulder pain (or more painful), opening it outward and raising it as high as possible, stretching the band - Simultaneously, rotate the hand on the side of the pain (or more painful) outward	LT, SA, and UT [62]
2nd	Bilateral elevation with shoulder external rotation	- Stand upright, looking straight ahead, and maintain a neutral position of the trunk and pelvis - Use both upper limbs - Bend both elbows until 90° and keep them close to your body - Position the hands slightly away from the midline, forming a diagonal with your body, with the thumbs pointing towards the ceiling	- While maintaining the position and distance between the two upper limbs, simultaneously raise both arms until the elbows reach shoulder height (90°)	- Repeat the steps of the exercise without resistance - Additionally, each hand should hold a part of the band, maintaining shoulder-width distance between them - Slightly move your hands away from the midline (about 30°), form a diagonal with your body, and keep the band stretched	- While maintaining the position and distance between the two upper limbs and keeping the band tensioned, simultaneously raise both arms until the elbows reach shoulder height (90°)	MT, LS, and LT [63]
	Shoulder external at 90° with scapular squeeze and trunk ipsilateral rotation without support	- Sit with knees bent at 90° and feet resting on the ground. - Look straight ahead and maintain a neutral, straight position of the trunk and pelvis with the assistance of abdominal contraction - Abduct and lift the arm on the side with shoulder pain (or more painful) laterally until the elbow reaches shoulder height (90°) - Bend the elbow on the side with shoulder pain (or more pain) to form a right angle (90°), with your hand pointing forward	- Rotate the trunk towards the side of the painful shoulder - Simultaneously, rotate your hand on the side with shoulder pain (or more painful) as much as possible upward - Squeeze both scapulae for 3 s	- Repeat the steps of the exercise without resistance - Additionally, position the hand of the limb that will not perform the exercise on the thigh to serve as a fixed point for the band - Hold the band with both hands, with a width ranging from the thigh on the side without pain (less painful) to the shoulder with pain (more painful) - Position the arm on the side with shoulder pain (or more painful) as follows: abduct and lift it laterally until the elbow reaches shoulder height (90°), and bend the elbow until 90°	- Rotate the trunk towards the side with shoulder pain (or more painful) - Simultaneously, rotate your hand on the side with shoulder pain (or more painful) as much as possible upward, pulling the band - Squeeze both scapulae for 3 s	MT, LT, and SA [61]
2nd	Prone scapular plane abduction	- Lie face down on a couch or bed, with a small towel supporting your forehead - Position the arm on the side with shoulder pain (or more painful) outside the couch/bed, hanging perpendicular to the floor, with the elbow extended - Let the opposite arm (without pain or less painful) relax and rest on the couch/bed	- Lift and open the upper limb on the side with shoulder pain (or more painful) until your arm is parallel to the floor and diagonal to your body, as if forming half of a Y shape	- Repeat the steps of the exercise without resistance - Additionally, hold one end of the band under your body to serve as a fixed point for the band - Hold the other end of the hand on the side with shoulder pain (or more painful), keeping the arm hanging towards the floor with the elbow extended	- Lift and open the upper limb on the side with shoulder pain (or more painful) until your arm is parallel to the floor and diagonal to your body, as if forming half of a Y shape, while stretching the band	LT and SA [64]
	Overhead height task	-	-	- Sit in front of a table and a box, with the top higher than the level of your head when seated - Bend your knees at a 90° angle and place your feet on the floor - Maintain a neutral position of the cervical, trunk, and pelvis - Ensure that the height of the table does not exceed the level of your chest - Place a half-liter (0.5 L) water bottle, for example, on the table [65]	- With the hand on the side with shoulder pain (or more painful), lift the bottle to the top of the box(es), ensuring it is higher than the level of the top of your head - After placing the bottle, return to the initial position by bringing the bottle back to its starting position	-

Legend: 1st—first intervention phase (week 1 to 4); 2nd—second intervention phase (week 5 to 8); 3 s—three seconds; flex-abd-ext.rot—flexion–abduction–external rotation; Interv.—intervention; LS—levator scapulae; LT—lower trapezius; Musc. Worked *—muscles recruited, at least to a moderate extent; MT—middle trapezius; SA—serratus anterior; UT—upper trapezius. Footnote: The sliding a box task (performed during the fourth week) and the overhead height task (performed during the eighth week) were added to increase global synergies with the whole upper limb and improve scapular function of assisting the hand interaction in space.

**Table A4:** Scapular and posterior capsule self-stretching exercises.

Exercise			
Name	Position		Description
	Anterior View	Posterior View	
**Pectoralis minor stretching**			Initial position [99]: - Stand upright; - Lift the arm on the side with shoulder pain (or more painful) until the elbow is at shoulder height and bent at 90°; - Place the hand against the wall. Movement [99]: - Maintaining the aforementioned position, lean your trunk forward and rotate it in the opposite direction of the arm with shoulder pain (or more painful).
**Upper trapezius and levator scapulae stretching**			Initial position [67,68]: - Seated; - Hold onto the chair seat with the hand on the side with shoulder pain (or more painful) to keep the shoulder down. Movement [67,68]: - Tilt your head to the opposite side (the side without pain or with less pain) and rotate it in that direction as well; - While maintaining this position, use the hand of the pain-free side (or less painful), bring your head down towards the axilla of the arm without pain in the shoulder (or less painful).
**Posterior shoulder stretching**			Initial position [69]: - Seated. Movement [69]: - Lift the working arm at shoulder height; - With the hand on the opposite side, press the elbow on the side with shoulder pain (or more painful) posteriorly.

Legend: 1st—first intervention phase (week 1 to 4); 2nd—second intervention phase (week 5 to 8); Interv.—intervention. Footnote: Stretching exercises were performed to enhance muscular flexibility and joint range of motion.
